# P-956. CASTing a Net: An Innovative Approach to Centralizing Antimicrobial Stewardship

**DOI:** 10.1093/ofid/ofaf695.1158

**Published:** 2026-01-11

**Authors:** Becca Nolen, Jessica Snawerdt, Dana Roth, Daniel Moussa, Pamela Andrews, Monica Cozad, Timmy Do

**Affiliations:** AdventHealth, Orlando, FL; AdventHealth, Orlando, FL; AdventHealth, Orlando, FL; AdventHealth, Orlando, FL; AdventHealth, Orlando, FL; AdventHealth, Orlando, FL; AdventHealth East Orlando, Orlando, FL

## Abstract

**Background:**

Infectious Diseases pharmacy expertise plays a pivotal role in delivering high-quality patient care and enhancing Antimicrobial Stewardship (AMS) programs. Multi-site healthcare systems with diverse resources often encounter challenges in maintaining consistent AMS efforts. Resource alignment and standardization provide a solution to these challenges to ensure goals are achieved.

**Figure:**
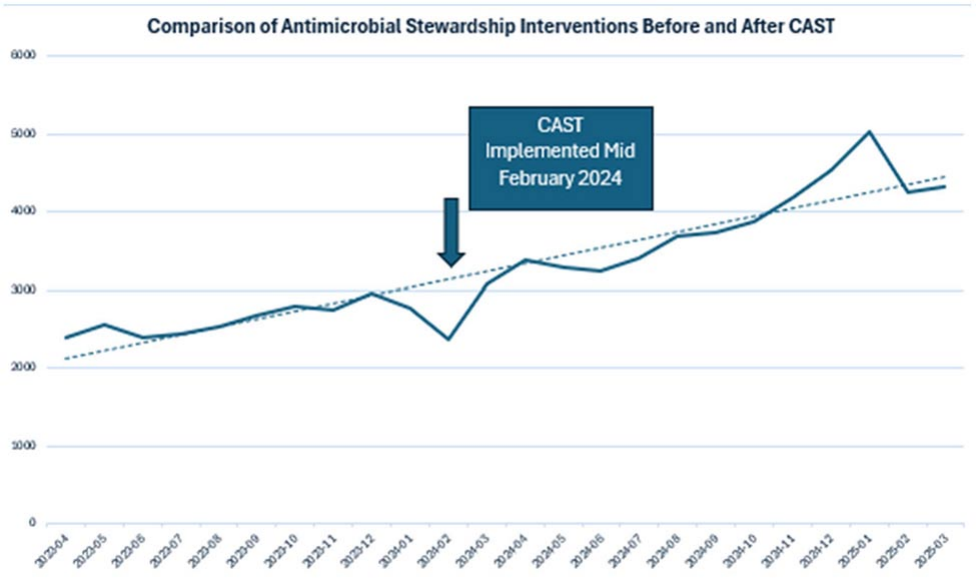

**Figure:**
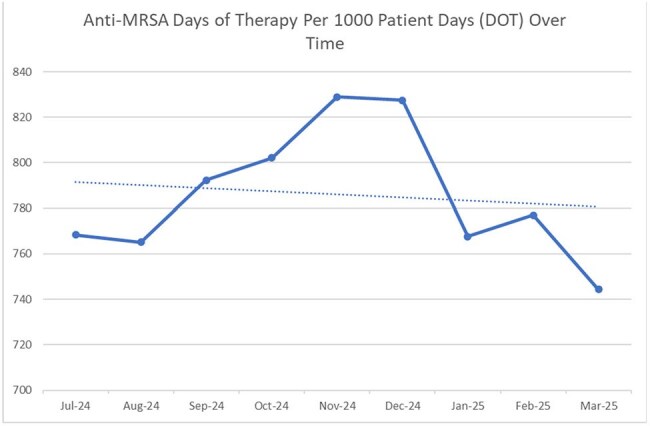

**Methods:**

In 2024, AdventHealth Central Florida Division (CFD), which consists of seven adult community hospitals and one tertiary referral center, implemented a Centralized Antimicrobial Stewardship Team (CAST) to standardize antimicrobial stewardship efforts at all adult hospitals. Two team members provide direct patient care exclusively at the tertiary referral center, while four team members manage patient care across the other seven campuses, with their time allocated based on bed capacity.

**Figure 3:**
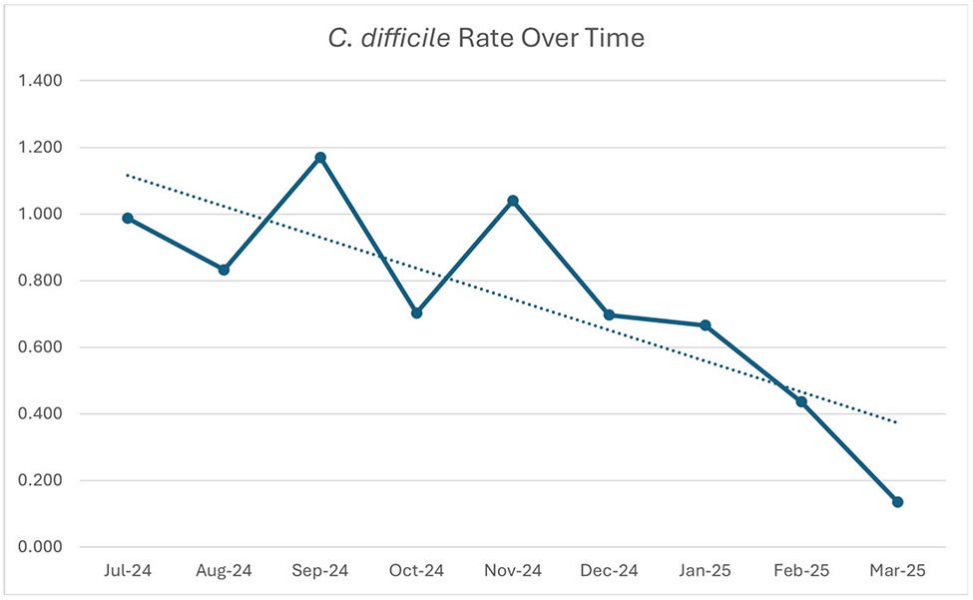

**Results:**

CAST consists of 7 Infectious Diseases pharmacists (including one clinical manager) that provide services to all adult CFD hospitals. Standardized clinical tasks include performing daily prospective audit and feedback, responding to high priority electronic health record (EHR) alerts, and reviewing blood cultures via integration with local pharmacy staff. Additionally, the team spearheads company-wide education, clinical initiatives, and formulary management of antimicrobials. The CAST model was introduced in February of 2024 with expansion to 7 members in August of 2024. Stewardship interventions increased by 50% compared to the period before CAST implementation (Figure 1). Additionally, anti-MRSA Days of Therapy (DOTs) have decreased by 23.96 since July (Figure 2) and *C. difficile* infection rates have decreased by 0.853 in that same period (Figure 3).

**Conclusion:**

A centralized antimicrobial stewardship model provides a scalable and effective approach to ensuring consistent antibiotic management in a large healthcare system with limited resources.

**Disclosures:**

All Authors: No reported disclosures

